# A Longitudinal Functional Neuroimaging Study in Medication-Naïve Depression after Antidepressant Treatment

**DOI:** 10.1371/journal.pone.0120828

**Published:** 2015-03-18

**Authors:** Hiroi Tomioka, Bun Yamagata, Shingo Kawasaki, Shenghong Pu, Akira Iwanami, Jinichi Hirano, Kazuyuki Nakagome, Masaru Mimura

**Affiliations:** 1 Department of Psychiatry, Showa University School of Medicine, Tokyo 157–8577, Japan; 2 Department of Neuropsychiatry, Keio University School of Medicine, Tokyo 160–8582, Japan; 3 Hitachi Medical Corporation, Application Development Office, Chiba 277–0804, Japan; 4 Division of Neuropsychiatry, Department of Brain and Neuroscience, Tottori University Faculty of Medicine, Tottori 683–8504, Japan; 5 National Center of Neurology and Psychiatry, Tokyo 187–8551, Japan

## Abstract

Recent studies have indicated the potential clinical use of near infrared spectroscopy (NIRS) as a tool in assisting the diagnosis of major depressive disorder (MDD); however, it is still unclear whether NIRS signal changes during cognitive task are state- or trait-dependent, and whether NIRS could be a neural predictor of treatment response. Therefore, we conducted a longitudinal study to explore frontal haemodynamic changes following antidepressant treatment in medication-naïve MDD using 52-channel NIRS. This study included 25 medication-naïve individuals with MDD and 62 healthy controls (HC). We performed NIRS scans before and after antidepressant treatment and measured changes of [oxy-Hb] activation during a verbal fluency task (VFT) following treatment. Individuals with MDD showed significantly decreased [oxy-Hb] values during a VFT compared with HC in the bilateral frontal and temporal cortices at baseline. There were no [oxy-Hb] changes between pre- and post-antidepressant treatment time points in the MDD cohort despite significant improvement in depressive symptoms. There was a significant association between mean [oxy-Hb] values during a VFT at baseline and improvement in depressive symptoms following treatment in the bilateral inferior frontal and middle temporal gyri in MDD. These findings suggest that hypofrontality response to a VFT may represent a potential trait marker for depression rather than a state marker. Moreover, the correlation analysis indicates that the NIRS signals before the initiation of treatment may be a biological marker to predict patient’s clinical response to antidepressant treatment. The present study provides further evidence to support a potential application of NIRS for the diagnosis and treatment of depression.

## Introduction

Major depressive disorder (MDD) has a high lifetime prevalence of up to 20% [1] and constitutes the leading cause of disability worldwide [2]. Neuroimaging techniques including positron emission tomography (PET), functional and structural magnetic resonance imaging (MRI) have been widely used to describe the potential neurobiological basis of MDD. Previous neuroimaging studies have revealed structural and functional aberrations in widely distributed brain regions, including the anterior cingulate cortex [3–5], orbitofrontal cortex [6,7], dorsolateral prefrontal cortex [8,9], amygdala [10], and hippocampus [5,6,8,10]. However, there has still been no established objective biomarker for the diagnosis and treatment for MDD.

Numerous studies using multi-channel near-infrared spectroscopy (NIRS), a noninvasive functional neuroimaging technique measuring the spatiotemporal characteristics of brain function, have consistently reported that oxygenated-hemoglobin [oxy-Hb] activation during a verbal fluency task (VFT) significantly decreased in patients with MDD compared with healthy controls (HC) in the fronto-temporal brain regions [11–14]. A recent meta-analysis of NIRS studies further supports previous findings of hypofrontality observed in MDD [15]. Moreover, a recent multi-site study with large samples found that frontal haemodynamic patterns detected by the NIRS method accurately distinguished between patients with MDD (74.6%) and those with the two other disorders (85.5%; bipolar disorder or schizophrenia) that presented with depressive symptoms [16]. These studies suggest that neuroimaging-guided differential diagnosis of major psychiatric disorders using NIRS may be a promising biomarker for personalizing care in clinical settings.

In Japan, NIRS has been used as a tool in assisting in the diagnosis of MDD, bipolar disorder, and schizophrenia with depressive symptoms as a clinical trial for several years; however, it is still unclear whether NIRS signal changes during VFT are state- or trait-dependent, the extent to which they are affected by antidepressant treatment, and whether NIRS could be a neural predictor of treatment response. Therefore, we performed a longitudinal study to explore the frontal haemodynamic changes following antidepressant treatment in medication-naïve MDD using 52-channel NIRS. Considering the very high rates of relapse in recovered subjects [17,18], we hypothesized that hypofrontality during the task would persist in remitted MDD subjects.

## Materials and Methods

### Participants

The patient group was comprised of 25 medication-naïve individuals with MDD and the HC group was comprised of 62 healthy individuals. All patients had a first-episode medication-naïve MDD. Participants with MDD were recruited from Showa University Hospital (Tokyo, Japan) and Tottori University Hospital (Tottori, Japan). Furthermore, HC individuals were recruited from the acquaintance of the authors and from the community through website advertisements. The participants with MDD were diagnosed by experienced psychiatrists (M.M. and K.N.) based on the criteria in the Diagnostic and Statistical Manual of Mental Disorders, 4th ed [19]. Participants were required to have had no lifetime history of bipolar disorder, psychosis, obsessive-compulsive disorder, or drug or alcohol misuse, as well as no neurological disorder. Furthermore, none of the participants reported unstable medical condition and history of significant head trauma. To rule out any psychiatric condition, experienced psychiatrists (M.M. and K.N.) examined all participants using the Mini-International Neuropsychiatric Interview [20,21]. Severity of depression was evaluated using the 17-item Hamilton Rating Scale for Depression (HRS-D) by trained psychiatrists who were involved in this study. Patients were required to have had depressive symptoms for at least 1month prior to enrollment and scores greater than 8 on the HRS-D at enrollment and baseline NIRS assessment. NIRS scans and HRS-D were acquired before starting antidepressant treatment (T1; baseline) and after 12 weeks of antidepressant treatment (T2) in the MDD group. Some (n = 6) of the patients were not able to come back for the second NIRS scan at the appointed time, therefore, their intervals between T1 and T2 were relatively longer. Patients started antidepressant treatment after the baseline NIRS scan (typically < 1 day). The patients were then treated with either paroxetine (20–40 mg/day), milnacipran (75–150 mg/day), or mirtazapine (30–45 mg/day). Partial remission was defined as ≤ 7 on the 17-item HRS-D based on DSM-IV criteria [19,22]. On the other hand, in HC group, we conducted NIRS scans and clinical evaluations only once at T1. After an extensive description of the study, written informed consent was obtained from all study participants. The study protocol was approved by the Ethics Committee of Showa University and Tottori University and prepared in accordance to the ethical standards of the Declaration of Helsinki.

### Verbal fluency task

Participants with MDD were measured twice at both T1 and T2. The task procedure in the present study was similar to that used by Takizawa and colleagues (2014) [16]. Participants sat in a comfortable chair and were instructed to relax and to avoid any major body movements, so as to avoid artifacts. Oxy-Hb changes were measured during VFT (letter version), which comprised of a 30s pre-task baseline, 60s VFT, and a 60s post-task baseline. For the pre- and post-task baseline periods, the participants were instructed to consecutively repeat the five Japanese vowels (“a”, “i”, “u”, “e”, “o”) aloud.

During the task period, participants were instructed to produce as many nouns as possible beginning with a designated syllable without the use of repetitions and proper nouns. The three sets of initial syllables (A;/to/, /se/, /o/, B; /a/, /ki/, /ha/, C; /na/, /i/, /ta/) were presented in counterbalanced order among the subjects and each syllable changed every 20s during the 60s task. The subtraction method (task minus pre- and post-task baseline) minimized the vocalization effects during VFT. The total number of correct words generated during VFT was adopted as a measure of task performance.

### NIRS measurements

We used a 52-channel NIRS machine (ETG-4000 Optical Topography System; Hitachi Medical Co., Japan) working with two different wavelengths (695 nm and 830 nm) and a time resolution of 10 Hz to measure relative changes of absorbed near-infrared light. These changes are transformed into concentration changes of [oxy-Hb], deoxygenated-hemoglobin [deoxy-Hb] and total-hemoglobin [total-Hb; sum of oxy-Hb and deoxy-Hb] as indicators for brain activity by means of a modified Beer–Lambert law [23]. The unit is mM×mm, i.e. changes of chromophore concentration depend on the path length of the near-infrared light.

We utilized 33 probes consisting of 17 light emitters and 16 detectors with an inter-optrode distance of 30 mm. A measuring point of activation (channel) was defined as the region between one emitter and one detector. Thus the probe set consisted of 52 channels and measures [Hb] in the bilateral prefrontal (approximately dorsolateral [Brodmann’s area (BA) 9, 46], ventrolateral [BA 44, 45, 47], frontopolar [BA 10]) and superior and middle temporal cortical surface regions. The panel was fastened to the head by elastic straps. The correspondence between the probe positions and the measurement areas on the cerebral cortex was confirmed based on a previous multi subject study of anatomical craniocerebral correction via the international 10–20 system [24,25].

The obtained data were analyzed using the “integral mode”; the pre-task baseline was determined as the mean over a 10s period just prior to the task period, and the post-task baseline was determined as the mean over the last 5s of the post-task period; linear fitting was applied to the data between these two baselines. A moving average method using a window width of 5s was applied to remove any short-term motion artifacts. Furthermore, we used a computer program that rejected a channel when artifact waveforms were visible [16].

For the analysis of the haemodynamic response data, Hb variables of each channel were averaged for the 60 s task period. We focused on [oxy-Hb] concentrations during the 60s task period, since [oxy-Hb] change was assumed to more directly reflect cognitive activation than [deoxy-Hb] change as shown by a stronger correlation with blood-oxygenation level-dependent (BOLD) signal measured by functional MRI (fMRI) [26].

### Data analysis

Regarding the behavioral data, we compared the mean numbers of produced words in VFT between groups at T1 using Student’s t-test. In NIRS data, first we compared the difference of mean activated [oxy-Hb] values during VFT for each channel between HC and MDD groups at T1 using Student’s t-test. Second, to assess the [oxy-Hb] response to antidepressant drug treatment for participants with MDD, we calculated the difference of mean [oxy-Hb] values during activation task for each channel between T1 and T2 in MDD group using two-tailed paired t-test. Third, to confirm whether mean [oxy-Hb] changes are state or trait dependent, Pearson correlation analysis between mean [oxy-Hb] changes during activation task for each channel and HRS-D changes between pre- and post-treatment was performed. We further conducted correlation analyses between mean [oxy-Hb] changes and difference of VFT scores between T1 and T2. Also, to explore the effect of antidepressants, correlation analysis was performed between the mean [oxy-Hb] changes between T1 and T2 and mean dose of antidepressants (Imipramine equivalent dose [mg]) at T2 in MDD patients. Finally, to investigate whether mean [oxy-Hb] values at baseline could become a potential predictor of treatment response, we performed Pearson correlation analyses between mean [oxy-Hb] values during VFT at T1 and HRS-D changes between pre- and post-treatment for each channel.

We adopted false discovery rate (FDR)-based procedure for the multiple testing correction in analyses using 52 channel data so that there are no more than 5% false positives on average (FDR-corrected) [27]. First, arranged the p value in ascending order, {P(1) ≤ P(2) ≤ P(3) … ≤ P(N)} corresponding to null hypotheses, {H(1), H(2), H(3), …, H(N)}. Second, evaluate the p value using Equation (1):

$$P\left(i\right)\leq i\times q/N$$ (1)

where q level is able to be selected in the range of zero to one. We used standard alpha level of 0.05. N is the total number of measurement channel (in this study, N = 52). Equation (1) starts from i = N in the reverse sequential order. Stop the comparison when the Equation (1) is true, choose at hypothesis H(k). Finally, reject all hypotheses {H(i)}_i = 1…k_ having p value which are less than or equal to P(k) and define the channels belonging to the rank i = 1…k are significant. Correlations were calculated with Pearson correlation coefficients. Statistical analyses were performed using SPSS 19.0J (IBM Inc., Armonk, NY) and MATLAB R2011 (MathWorks Inc., Massachusetts, US) software.

## Results

### Demographic characteristics

There were no significant differences in age, gender ratio, and VFT performance between HC and MDD groups at T1. While VFT scores were not significantly different in MDD between T1 and T2, the MDD group demonstrated significant improvement of depressive symptoms in HRS-D after antidepressant treatment (Table 1). Seventeen of 25 participants with MDD partially remitted after antidepressant drug treatment (the HRS-D ≤ 7).

**Table 1:** Demographic and clinical characteristics.

	MDD		HC	*t*-test (p value)	
	T1	T2		T1 vs T2	HC vs MDD (T1)
n	25	-	62	-	-
Age, years	51.9 ± 16.6	-	51.7 ± 17.2	-	0.97
Gender, women/men	22 / 3	-	48 / 14	-	0.74
Age at onset, years	50.2 ± 17.9	-	-	-	-
Duration of illness, weeks	25.0 ± 30.0	-	-	-	-
NIRS measurement period, weeks	13.2 ± 13.0	-	-	-	-
VFT	13.2 ± 4.4	13.5 ± 4.7	12.8 ± 4.2	0.79	0.66
VFT (remitted MDD)	13.4 ± 3.3	14.6 ± 3.9	-	0.16	-
HRS-D	19.0 ± 6.6	6.5 ± 5.8	-	<0.01	-
Imipramine equivalent dose, mg	0.0 ± 0.0	118.7 ± 67.3	-	<0.01	-

NIRS scans and behavioral data were acquired before starting an antidepressant treatment (T1) and after treatment (T2). MDD, Major depressive disorder; HC, Healthy controls; NIRS, Near-infrared spectroscopy; VFT, Verbal fluency task; HRS-D, 17-item Hamilton rating scale for Depression.

### Group comparison at baseline measure

**Fig 1:**
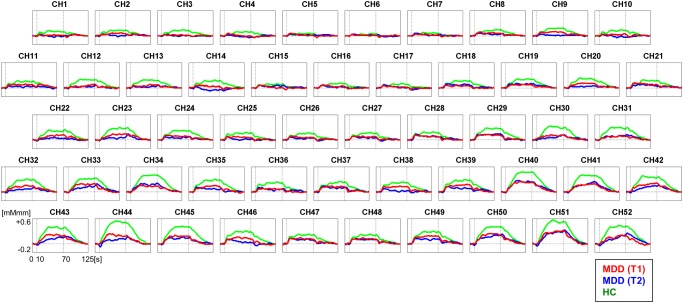
Grand average waveforms of [oxy-Hb] changes during VFT in the fronto-temporal brain regions. Red line represents MDD (T1), blue line represents MDD (T2), and green line represents HC (T1). T1: baseline, T2: after treatment. oxy-Hb, oxygenated haemoglobin signal; VFT, Verbal fluency task, MDD, Major depressive disorder; HC, Healthy controls.

**Fig 2:**
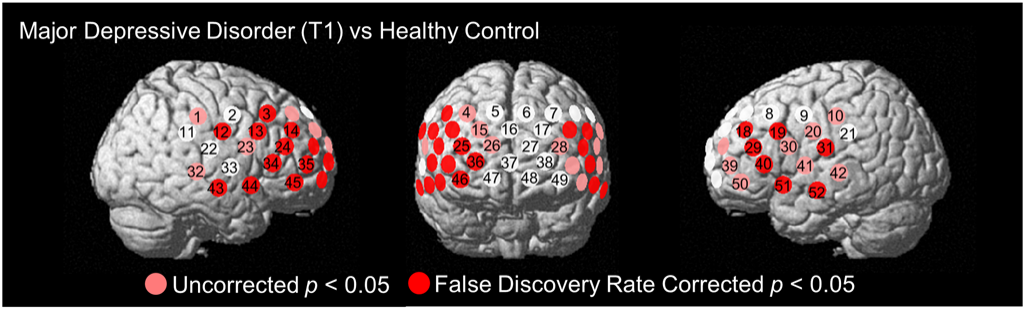
Results of comparison of mean [oxy-Hb] values during VFT between groups at T1 (MDD vs HC). Channels in red showed significantly decreased [oxy-Hb] activations during VFT in MDD compared with HC (*P* < 0.05; FDR-corrected). T1: baseline.

Mean [oxy-Hb] values activated by VFT were significantly reduced in participants with MDD compared with HC individuals at 20 channels (ch3, 12–14, 18, 19, 24, 25, 29, 31, 34–36, 40, 43–46, 51–52; FDR-corrected *P* = 0.003 to 0.016) located in the bilateral prefrontal and temporal brain regions at T1 (Fig. 1 and Fig. 2).

### Longitudinal comparison of mean [oxy-Hb] values between pre- and post-treatment

There were no significant differences in mean [oxy-Hb] values during VFT between T1 and T2 for all 52 channels in MDD (FDR-corrected) (Fig. 1).

**Fig 3:**
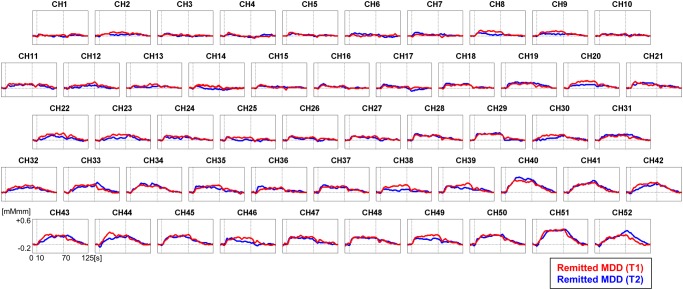
Grand average waveforms of [oxy-Hb] changes during VFT in the fronto-temporal brain regions in remitted MDD. Red line represents remitted MDD (T1: baseline) and blue line represents remitted MDD (T2: after treatment). oxy-Hb, oxygenated haemoglobin signal; VFT, Verbal fluency task; MDD, Major depressive disorder.

When analyzing individuals with partially remitted MDD (n = 17), we obtained the same results such that there were no differences in mean [oxy-Hb] values during VFT between pre- and post-treatments for all 52 channels (Fig. 3).

### Relationship between mean [oxy-Hb] values and clinical response to treatment

Mean [oxy-Hb] values during VFT were not significantly correlated with HRS-D scores in all 52 channels at T1 and T2, respectively. There was no significant correlation between the changes of mean [oxy-Hb] values during VFT and HRS-D scores between T1 and T2 in any channels. In addition, there was no significant correlation between mean [oxy-Hb] changes between T1 and T2 and mean dose of antidepressants at T2. Moreover, mean [oxy-Hb] changes from T1 to T2 did not show any significant correlations with the difference of VFT scores between T1 and T2.

**Fig 4:**
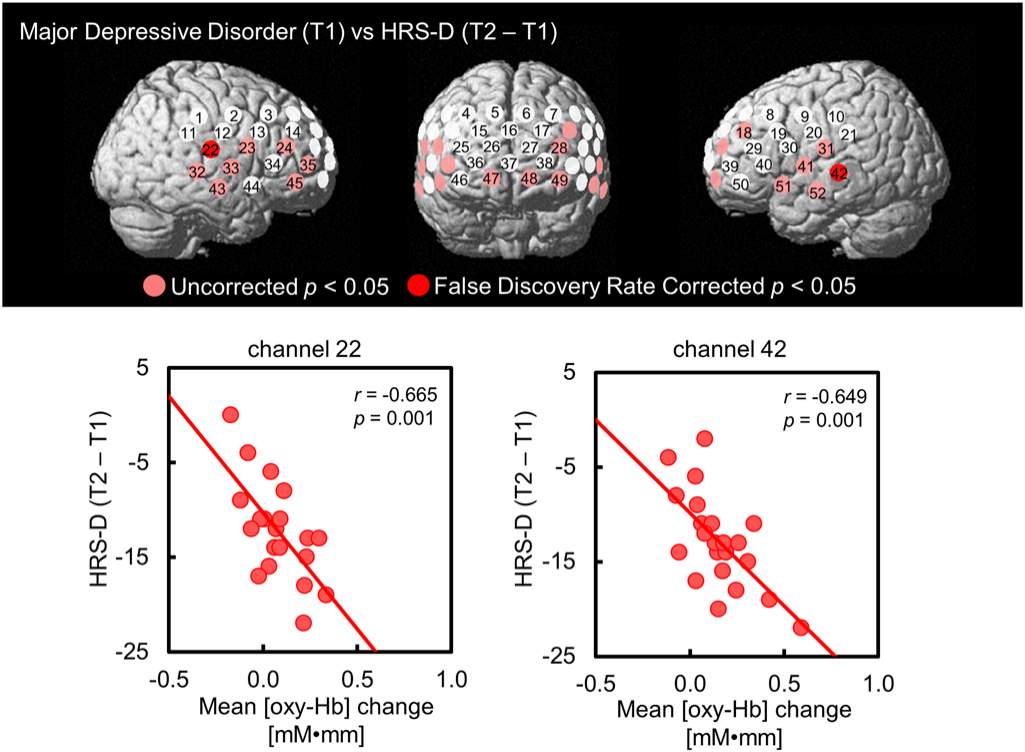
Correlations between mean [oxy-Hb] values and antidepressant treatment. Correlation of mean [oxy-Hb] values during VFT at T1 with changes of HRS-D scores following antidepressant treatment (T2—T1) in MDD. T1: baseline, T2: after treatment. Above: Channels in red (ch 22 and 42) showed a significant correlation (*P* < 0.05; FDR-corrected) and channels in pink also showed a significant correlation (*P* < 0.05; uncorrected). Below: Scatterplots for correlations in ch 22 and 42. oxy-Hb, oxygenated haemoglobin signal; VFT, Verbal fluency task; HRS-D, 17-item Hamilton rating scale for Depression; MDD, Major depressive disorder.

There was a significant association between mean [oxy-Hb] values during VFT at T1 and changes in HRS-D scores from T1 to T2 in the bilateral inferior frontal gyrus and middle temporal gyrus including ch22 (*r* = -0.665; FDR-corrected *P* = 0.001), and ch 42 (*r* = -0.649; FDR-corrected *P* = 0.001) (Fig. 4). This association was such that MDD patients who showed greater baseline [oxy-Hb] activation during VFT in the inferior frontal and middle temporal regions tend to show greater improvement in depressive symptoms after treatment. When focusing on the partially remitted MDD group (n = 17), we found significant negative correlations between mean [oxy-Hb] changes during VFT at T1 and changes in HRS-D scores from T1 to T2 in 10 channels (ch 20, 22, 23, 31, 32, 33, 35, 42, 51, 52; uncorrected *P* = 0.002 to 0.040); however, there were no significant channels with 52-channel FDR-correction.

We further conducted multiple regression analysis (stepwise method; entry: *P* = 0.05 and removal: *P* = 0.10) to confirm the associations between mean [oxy-Hb] values during VFT at T1 and other confounding clinical factors at ch22 and ch42. Therefore, mean [oxy-Hb] values during VFT at T1 were adopted as a dependent variable and VFT scores at T1, VFT scores at T2, HRS-D scores at T1, HRS-D scores at T2, changes of HRS-D scores from T1 to T2, and imipramine dose at T2 were adopted as independent variables. As a result, significant associations were only observed between mean [oxy-Hb] values during VFT at T1 and changes in HRS-D scores from T1 to T2 (ch22: *R* ^*2*^ = 0.442, adjusted *R* ^*2*^ = 0.411, beta = -0.665, *P* = 0.001; ch42: *R* ^*2*^ = 0.421, adjusted *R* ^*2*^ = 0.392, beta = -0.649, *P* = 0.001) after controlling for other potential confounding factors.

## Discussion

We performed a longitudinal study to investigate the effects of antidepressant treatment on brain activation using functional NIRS in individuals with MDD. This study revealed that smaller frontal [oxy-Hb] activation during VFT in medication-naïve MDD compared with HC were not significantly different between before and after antidepressant treatments despite significant improvement in HRS-D scores. Moreover, there was no association between mean [oxy-Hb] changes and the improvement in HRS-D scores between pre- and post-treatment period. The lack of significant correlation between mean [oxy-Hb] changes between pre- and post-treatment and mean dose of antidepressants (at T2) indicates that the above findings cannot solely be explained by the effect of antidepressant drugs. These findings mean that hypofrontality observed in MDD remained unchanged even after appropriate pharmacotherapy and overall improvement of depressive symptoms. Furthermore, we found the baseline mean [oxy-Hb] values during VFT demonstrated significant correlations with the HRS-D changes after antidepressant treatment in the bilateral inferior frontal and left middle temporal gyri. This finding may indicate that NIRS measurement before treatment may be a potential predictor of treatment response in medication-naïve MDD.

In the group comparison, the present study showed mean [oxy-Hb] activation during VFT to be significantly smaller in the MDD group than in age- and gender-matched HC in prefrontal and temporal cortices. This result is consistent with those previous NIRS studies [11–14]. Therefore, our findings of hypofrontality during VFT in medication-naïve MDD further support these previous studies and indicate the validity of our findings.

As hypothesized, our longitudinal study showed that hypoactivation during VFT in the bilateral prefrontal and temporal cortices essentially remained unchanged even after improvement of depressive symptoms. Our findings are consistent with a previous fMRI study using VFT investigating changes of brain activation in remitted patients with depression [28]. The authors found that even in remitted patients, there was reduced activation in the left prefrontal cortex compared to healthy controls. This finding may indicate that, within this verbal fluency neural network, brain function in the left prefrontal cortex remains impaired in remitted patients despite clear clinical improvements. In addition, several recent fMRI studies using emotional tasks in remitted depression have consistently reported that even in the absence of current symptoms, individuals with remitted depression showed reduced activity in brain regions related to the emotion network compared to healthy controls and suggest that these brain dysfunctions may be a specific trait maker for depression [29–31].

Moreover it has been reported that MDD have very high rates of relapse. Previous studies have shown that more than 50% of patients who recovered from an initial episode of depression will relapse at least once, and this figure increases to 70% and 80% for those who have had two or more depressive episodes [18]. These findings indicate that there may exist some latent neural vulnerabilities in remitted MDD subjects. Therefore, our findings of persistent functional abnormality in the bilateral prefrontal cortex from before to after antidepressant treatment may be a trait maker or neurobiological vulnerability for depression rather than a state maker.

On the other hand, a recent study showed that the frontopolar [oxy-Hb] increase following six weeks of psychodynamic therapy in 10 children with depression (mean age: 12.9 ± 0.9 years) using two-channel NIRS and suggested the frontopolar response may be a state maker for depressive mood in children with depression [32]. This inconsistency may be caused by differences in the NIRS measuring method, sample heterogeneity, and their pathophysiology. They did not measure [oxy-Hb] concentration of the entire frontal cortex due to using two-channel NIRS. Moreover, even after excluding mental retardation, pervasive developmental disorders, eating disorders, attention deficit/hyperactivity disorders, or oppositional defiant disorders from the sample, it is possible the sample still included bipolar disorder because of a high rate of undiagnosed bipolar disorder among prepubertal children with MDD [33–35]. Furthermore, previous neuroimaging researches have indicated different pathophysiology between children and adults with MDD, possibly reflecting ongoing neuroplastic changes and the impact of many years depression on neural connectivity [36,37]. In addition, our findings are not in line with previous PET and fMRI studies reporting that antidepressant treatments tend to restore normal brain function [38–40], while improving depressive symptoms. However, these previous studies have produced somewhat inconsistent results. For example, meta-analyses of PET and fMRI studies following antidepressant treatment have found that activity in a variety of prefrontal cortex, thalamic, and insular areas increase during treatment, whereas activity in the amygdala, hippocampus, ventral anterior cingulate cortex, and other prefrontal areas appear to decrease during treatment [38,41]. These discrepancies may have been caused by differences across brain imaging methodologies and activation paradigms including cognitive or emotional tasks (e.g. either a resting state or one of several task activation paradigms). Furthermore, in the present study we adopted VFT for activation whereas most previous longitudinal fMRI studies utilized emotional tasks and to our knowledge, there have been no longitudinal fMRI studies using VFT in medication-naïve depression. Therefore, we are not able to directly compare our findings with other previous neuroimaging studies.

Taken together, our results may indicate that NIRS is a more specific tool as an adjunct to the diagnosis of depression compared with other brain imaging techniques due to our hypothesis that NIRS signal represents a potential trait marker for depression rather than a state marker.

The other main purpose of the present study was to examine neural predictors of treatment response. One of the most highly replicated observations in longitudinal fMRI studies with emotional task in depression has been the relationship of anterior cingulate cortical activity during an acute depressive episode and clinical response after treatment [42]. In this study, we also found a significant negative correlation between the mean [oxy-Hb] values during VFT at baseline and the HRS-D score changes after treatment in the bilateral inferior frontal and left middle temporal gyri, which means the greater the mean [oxy-Hb] activation before treatment, the greater clinical improvement after therapy. The location of our findings was consistent with previous neuroimaging studies indicating that initial activations in the inferior frontal [43] and middle temporal gyri [44] may be a neural predictor of clinical response in the treatment of depression. Our correlation results may suggest that predictive association of mean [oxy-Hb] values at baseline and treatment response in unmedicated depression.

To our knowledge, this is the first longitudinal NIRS study to investigate the effects of antidepressant treatment on brain activation in adults with medication-naïve depression. The advantage of this study is using the participants who have never taken any antidepressant drugs at the initial measurement. Thus, this study excluded confounding effects of continuous antidepressant use on prefrontal cortex function at the initial measurement and allowed us to observe pure treatment responses in depression. On the other hand, several limitations of this study should also be noted here. First, since we did not repeat the NIRS measurements in healthy controls, we compared group differences at baseline and then performed a paired t-test in MDD group instead of conducting 2 x 2 (diagnosis [depression, controls] × treatment interval [T1, T2]) analyses of variance (ANOVA). However a previous NIRS study examined short- and long-term retest reliability of brain activity during VFT in healthy subjects and demonstrated reasonable retest reliability with intervals of three weeks and one year [45]. Hence, we may be able to assume the [oxy-Hb] data of controls after treatment are statistically similar with those at baseline. Second, there may be a possibility of taking a longer time to normalize brain activity than the observed clinical improvement in depression. Even though our 13-week interval is relatively longer than other longitudinal studies because it is common for studies to follow patients with MDD for 8 weeks or less, mean [oxy-Hb] changes after treatment were still not observed in this study. Therefore, a longitudinal study with an even longer interval for both depression and controls is needed.

In summary, our study revealed potentially important abnormalities of frontal activation in response to a VFT in depression. Even though depressive symptoms significantly improved after antidepressant treatment, remitted depression showed persistent hypofrontality during a VFT. These findings suggest that aberrant prefrontal cortex response to a VFT may represent a potential trait maker for depression. Moreover, our findings from the correlation analyses suggest that the NIRS data before the initiation of treatment may play a key role as a biological marker to predict patient’s clinical response to antidepressant treatment. Together with its safety, low cost, portability, and high temporal resolution, the present study provides further evidence to support the potential clinical application of NIRS in practical psychiatric settings.

## References

[1] Kessler RC, Chiu WT, Demler O, Merikangas KR, Walters EE. Prevalence, severity, and comorbidity of 12-month DSM-IV disorders in the National Comorbidity Survey Replication. Arch Gen Psychiatry. 2005;62: 617–627. 15939839 10.1001/archpsyc.62.6.617 PMC2847357

[2] Zhang J, Wang J, Wu Q, Kuang W, Huang X, He Y, et al Disrupted brain connectivity networks in drug-naive, first-episode major depressive disorder. Biol Psychiatry. 2011;70: 334–342. 10.1016/j.biopsych.2011.05.018 21791259

[3] Bora E, Harrison BJ, Davey CG, Yucel M, Pantelis C. Meta-analysis of volumetric abnormalities in cortico-striatal-pallidal-thalamic circuits in major depressive disorder. Psychol Med. 2012;42: 671–681. 10.1017/S0033291711001668 21910935

[4] Drevets WC, Bogers W, Raichle ME. Functional anatomical correlates of antidepressant drug treatment assessed using PET measures of regional glucose metabolism. Eur Neuropsychopharmacol. 2002;12: 527–544. 12468016 10.1016/s0924-977x(02)00102-5

[5] Du MY, Wu QZ, Yue Q, Li J, Liao Y, Kuang WH, et al Voxelwise meta-analysis of gray matter reduction in major depressive disorder. Prog Neuropsychopharmacol Biol Psychiatry. 2012;36: 11–16. 10.1016/j.pnpbp.2011.09.014 22001316

[6] Peng J, Liu J, Nie B, Li Y, Shan B, Wang G, et al Cerebral and cerebellar gray matter reduction in first-episode patients with major depressive disorder: a voxel-based morphometry study. Eur J Radiol. 2011;80: 395–399. 10.1016/j.ejrad.2010.04.006 20466498

[7] Wagner G, Koch K, Schachtzabel C, Reichenbach JR, Sauer H, Schlösser Md RG. Enhanced rostral anterior cingulate cortex activation during cognitive control is related to orbitofrontal volume reduction in unipolar depression. J Psychiatry Neurosci. 2008;33: 199–208. 18592043 PMC2441889

[8] Ritchey M, Dolcos F, Eddington KM, Strauman TJ, Cabeza R. Neural correlates of emotional processing in depression: changes with cognitive behavioral therapy and predictors of treatment response. J Psychiatr Res. 2011;45: 577–587. 10.1016/j.jpsychires.2010.09.007 20934190 PMC3042483

[9] Zhong M, Wang X, Xiao J, Yi J, Zhu X, Liao J, et al Amygdala hyperactivation and prefrontal hypoactivation in subjects with cognitive vulnerability to depression. Biol Psychol. 2011;88: 233–242. 10.1016/j.biopsycho.2011.08.007 21878364

[10] Lee HY, Tae WS, Yoon HK, Lee BT, Paik JW, Son KR, et al Demonstration of decreased gray matter concentration in the midbrain encompassing the dorsal raphe nucleus and the limbic subcortical regions in major depressive disorder: an optimized voxel-based morphometry study. J Affect Disord. 2011;133: 128–136. 10.1016/j.jad.2011.04.006 21546094

[11] Kameyama M, Fukuda M, Yamagishi Y, Sato T, Uehara T, Ito M, et al Frontal lobe function in bipolar disorder: a multichannel near-infrared spectroscopy study. Neuroimage. 2006;29: 172–184. 16125979 10.1016/j.neuroimage.2005.07.025

[12] Matsuo K, Kato N, Kato T. Decreased cerebral haemodynamic response to cognitive and physiological tasks in mood disorders as shown by near-infrared spectroscopy. Psychol Med. 2002;32: 1029–1037. 12214784 10.1017/s0033291702005974

[13] Pu S, Matsumura H, Yamada T, Ikezawa S, Mitani H, Adachi A, et al Reduced frontopolar activation during verbal fluency task associated with poor social functioning in late-onset major depression: Multi-channel near-infrared spectroscopy study. Psychiatry Clin Neurosci. 2008;62: 728–737. 10.1111/j.1440-1819.2008.01882.x 19068011

[14] Suto T, Fukuda M, Ito M, Uehara T, Mikuni M. Multichannel near-infrared spectroscopy in depression and schizophrenia: cognitive brain activation study. Biol Psychiatry. 2004;55: 501–511. 15023578 10.1016/j.biopsych.2003.09.008

[15] Zhang H, Dong W, Dang W, Quan W, Tian J, Chen R, et al Near-infrared spectroscopy for examination of prefrontal activation during cognitive tasks in patients with major depressive disorder: a meta-analysis of observational studies. Psychiat Clin Neurosci. 2015;69; 22–33. 10.1111/pcn.12209 24897940

[16] Takizawa R, Fukuda M, Kawasaki S, Kasai K, Mimura M, Pu S, et al Neuroimaging-aided differential diagnosis of the depressive state. Neuroimage. 2014;85 Pt 1: 498–507. 10.1016/j.neuroimage.2013.05.126 23764293

[17] Consensus Development Panel. NIMH/NIH Consensus Development Conference statement. Mood disorders: pharmacologic prevention of recurrences. Consensus Development Panel. Am J Psychiatry. 1985;142: 469–476. 3976921 10.1176/ajp.142.4.469

[18] Shea MT, Elkin I, Imber SD, Sotsky SM, Watkins JT, Collins JF, et al Course of depressive symptoms over follow-up. Findings from the National Institute of Mental Health Treatment of Depression Collaborative Research Program. Arch Gen Psychiatry. 1992;49: 782–787. 1417430 10.1001/archpsyc.1992.01820100026006

[19] American Psychiatric Association Diagnostic and Statistical Manual of Mental Disorders IV Edition Text Revision (DSM-IV-TR). Washington DC: APA; 2000.

[20] Otsubo T, Tanaka K, Koda R, Shinoda J, Sano N, Tanaka S, et al Reliability and validity of Japanese version of the Mini-International Neuropsychiatric Interview. Psychiatry Clin Neurosci. 2005;59: 517–26. 16194252 10.1111/j.1440-1819.2005.01408.x

[21] Sheehan DV, Lecrubier Y, Sheehan KH, Amorim P, Janavs J, Weiller E, et al The Mini-International Neuropsychiatric Interview (M.I.N.I.): the development and validation of a structured diagnostic psychiatric interview for DSM-IV and ICD-10. J Clin Psychiatry. 59 Suppl. 1998;20: 22–33. 9881538

[22] Frank E, Prien RF, Jarrett RB, Keller MB, Kupfer DJ, Lavori PW, et al Conceptualization and rationale for consensus definitions of terms in major depressive disorder. Remission, recovery, relapse, and recurrence. Arch Gen Psychiatry. 1991;48: 851–855. 1929776 10.1001/archpsyc.1991.01810330075011

[23] Obrig H, Villringer A. Beyond the visible—imaging the human brain with light. J Cereb Blood Flow Metab. 2003;23: 1–18. 12500086 10.1097/01.WCB.0000043472.45775.29

[24] Okamoto M, Dan H, Sakamoto K, Takeo K, Shimizu K, Kohno S, et al Three-dimensional probabilistic anatomical cranio-cerebral correlation via the international 10–20 system oriented for transcranial functional brain mapping. NeuroImage. 2004;21: 99–111. 14741647 10.1016/j.neuroimage.2003.08.026

[25] Tsuzuki D, Jurcak V, Singh AK, Okamoto M, Watanabe E, Dan I. Virtual spatial registration of stand-alone fNIRS data to MNI space. Neuroimage. 2007;34: 1506–1518. 17207638 10.1016/j.neuroimage.2006.10.043

[26] Strangman G, Culver JP, Thompson JH, Boas DA. A quantitative comparison of simultaneous BOLD fMRI and NIRS recordings during functional brain activation. Neuroimage. 2002;17: 719–731. 12377147

[27] Benjamini Y, Hochberg Y. Controlling the false discovery rate: a practical and powerful approach to multiple testing. J R Statist Soc Ser B. 1995;57: 289–300.

[28] Okada G, Okamoto Y, Yamashita H, Ueda K, Takami H, Yamawaki S. Attenuated prefrontal activation during a verbal fluency task in remitted major depression. Psychiatry Clin Neurosci. 2009;63: 423–425. 10.1111/j.1440-1819.2009.01952.x 19566776

[29] Elliott R, Lythe K, Lee R, McKie S, Juhasz G, Thomas EJ, et al Reduced medial prefrontal responses to social interaction images in remitted depression. Arch Gen Psychiatry. 2012;69: 37–45. 10.1001/archgenpsychiatry.2011.139 22213787

[30] Kanske P, Heissler J, Schonfelder S, Wessa M. Neural correlates of emotion regulation deficits in remitted depression: the influence of regulation strategy, habitual regulation use, and emotional valence. Neuroimage. 2012;61: 686–693. 10.1016/j.neuroimage.2012.03.089 22613776

[31] Smoski MJ, Keng SL, Schiller CE, Minkel J, Dichter GS. Neural mechanisms of cognitive reappraisal in remitted major depressive disorder. J Affect Disord. 2013;151: 171–177. 10.1016/j.jad.2013.05.073 23796796 PMC3769423

[32] Usami M, Iwadare Y, Kodaira M, Watanabe K, Saito K. Near infrared spectroscopy study of the frontopolar hemodynamic response and depressive mood in children with major depressive disorder: a pilot study. PLoS One 2014;9: e86290 10.1371/journal.pone.0086290 24466008 PMC3900510

[33] Geller B, Fox LW, Clark KA. Rate and predictors of prepubertal bipolarity during follow-up of 6- to 12-year-old depressed children. J Am Acad Child Adolesc Psychiatry. 1994;33: 461–468. 8005898 10.1097/00004583-199405000-00003

[34] Geller B, Zimerman B, Williams M, Bolhofner K, Craney JL. Bipolar disorder at prospective follow-up of adults who had prepubertal major depressive disorder. Am J Psychiatry. 2001;158: 125–127. 11136645 10.1176/appi.ajp.158.1.125

[35] Rao U, Ryan ND, Birmaher B, Dahl RE, Williamson DE, Kaufman J, et al Unipolar depression in adolescents: clinical outcome in adulthood. J Am Acad Child Adolesc Psychiatry. 1995;34: 566–578. 7775352 10.1097/00004583-199505000-00009

[36] Hulvershorn LA, Cullen K, Anand A. Toward dysfunctional connectivity: a review of neuroimaging findings in pediatric major depressive disorder. Brain Imaging Behav. 2011;5: 307–328. 10.1007/s11682-011-9134-3 21901425 PMC3216118

[37] Rao U, Chen LA. Characteristics, correlates, and outcomes of childhood and adolescent depressive disorders. Dialogues Clin Neurosci. 2009;11: 45–62. 19432387 10.31887/DCNS.2009.11.1/urao PMC2766280

[38] Fitzgerald PB, Laird AR, Maller J, Daskalakis ZJ. A meta-analytic study of changes in brain activation in depression. Hum Brain Mapp. 2008;29: 683–695. 17598168 10.1002/hbm.20426 PMC2873772

[39] Mayberg HS. Modulating dysfunctional limbic-cortical circuits in depression: towards development of brain-based algorithms for diagnosis and optimised treatment. Br Med Bull. 2003;65: 193–207. 12697626 10.1093/bmb/65.1.193

[40] Mayberg HS, Brannan SK, Tekell JL, Silva JA, Mahurin RK, et al (2000) Regional metabolic effects of fluoxetine in major depression: serial changes and relationship to clinical response. Biol Psychiatry. 2000;48: 830–843. 11063978 10.1016/s0006-3223(00)01036-2

[41] Delaveau P, Jabourian M, Lemogne C, Guionnet S, Bergouignan L, Fossati P. Brain effects of antidepressants in major depression: a meta-analysis of emotional processing studies. J Affect Disord. 2011;130: 66–74. 10.1016/j.jad.2010.09.032 21030092

[42] Fu CHY, Walsh ND, Drevets WC. Neuroimaging studies of mood disorders In: CHY Fu T Russell C Senior DR Weinberger RM Murray, editors. A Guide to Neuroimaging in Psychiatry. London: Martin Dunitz; 2003 pp. 131–169.

[43] Walsh ND, Williams SC, Brammer MJ, Bullmore ET, Kim J, Suckling J, et al A longitudinal functional magnetic resonance imaging study of verbal working memory in depression after antidepressant therapy. Biol Psychiatry. 2007;62: 1236–1243. 17601497 10.1016/j.biopsych.2006.12.022

[44] Heller AS, Johnstone T, Peterson MJ, Kolden GG, Kalin NH, Davidson RJ. Increased prefrontal cortex activity during negative emotion regulation as a predictor of depression symptom severity trajectory over 6 months. JAMA Psychiatry. 2013;70: 1181–1189. 10.1001/jamapsychiatry.2013.2430 24173657 PMC3866958

[45] Schecklmann M, Ehlis AC, Plichta MM, Fallgatter AJ. Functional near-infrared spectroscopy: a long-term reliable tool for measuring brain activity during verbal fluency. Neuroimage. 2008;43: 147–155. 10.1016/j.neuroimage.2008.06.032 18657624

